# Prolonged Neuromuscular Paralysis Following Succinylcholine Induction Leading to a Trans-Kambin Oblique Lateral Lumbar Interbody Fusion (OLLIF) Procedure Performed Without Neuromonitoring

**DOI:** 10.7759/cureus.105797

**Published:** 2026-03-24

**Authors:** Hamid Abbasi, Abdul Rauf, Dominic Moore

**Affiliations:** 1 Spine Surgery, Avicenna Technical University, Burnsville, USA; 2 Spine Surgery, Inspired Spine, Burnsville, USA; 3 Neurosurgery, Inspired Spine, Burnsville, USA

**Keywords:** degenerative disc disease, intraoperative neuromonitoring, lumbar fusion, minimally invasive spine surgery, neuromuscular blockade, oblique lateral lumbar interbody fusion, pharmacogenetic variability, pseudocholinesterase deficiency, succinylcholine-induced paralysis, trans-kambin ollif

## Abstract

Intraoperative neuromonitoring (IONM) is widely used during minimally invasive spine procedures, including trans-Kambin oblique lateral lumbar interbody fusion, to reduce the risk of neural injury. We present a unique case in which prolonged neuromuscular paralysis following succinylcholine induction resulted in the absence of effective neuromonitoring. After careful multidisciplinary consideration of case cancellation and a detailed risk-benefit assessment, the procedure was completed because it involved a single level without complicating anatomical factors. The patient experienced an excellent clinical outcome with no postoperative neurological deficit. This case highlights important anesthetic considerations, potential pharmacogenetic variability, and nuanced intraoperative decision-making when neuromonitoring becomes unexpectedly unavailable.

## Introduction

Trans-Kambin oblique lateral lumbar interbody fusion (OLLIF) is a minimally invasive lumbar fusion technique that accesses the disc space through Kambin’s triangle while minimizing posterior muscle disruption and tissue trauma [1-4]. Because of the proximity to neural elements, intraoperative neuromonitoring (IONM) is commonly employed to enhance safety during instrumentation and cage placement. Modalities such as free-run electromyography (EMG), triggered EMG, and motor evoked potentials (MEPs) are routinely used during trans-Kambin approaches to detect impending neural compromise [5,6].

Neuromuscular blocking agents are frequently used during anesthesia induction to facilitate intubation and optimize surgical conditions. Succinylcholine is favored for its rapid onset and short duration of action; however, rare cases of prolonged neuromuscular paralysis have been described, most commonly related to pseudocholinesterase deficiency or genetic variability affecting drug metabolism [7,8]. When prolonged paralysis occurs, the reliability or availability of neuromonitoring may be significantly impaired, presenting an unexpected intraoperative challenge. This report describes such a scenario and discusses its implications.

IONM in spine surgery

IONM is increasingly utilized in spine surgery to reduce the risk of iatrogenic neurological injury, particularly during complex or high-risk procedures [5,6]. National data from the United States demonstrate that, although neuromonitoring use has increased over time, it is not universal. Overall utilization across spine surgeries rose from approximately 1% in 2007 to about 12% in 2011, with higher rates reported for deformity surgery compared with routine degenerative procedures [5]. In the context of trans-Kambin approaches, neuromonitoring, particularly triggered and free-run EMG, plays a critical role in providing real-time feedback on nerve root proximity during dilation and cage placement, thereby enhancing procedural safety.

More recent analyses continue to demonstrate variability in utilization patterns. Large database studies evaluating cost-effectiveness and utilization trends have shown that neuromonitoring is most frequently employed in complex procedures such as deformity correction, tumor surgery, and cervical or thoracic operations involving the spinal cord [5]. In contrast, routine lumbar degenerative procedures, particularly single-level fusions, are more commonly performed without neuromonitoring.

Practice patterns vary considerably by surgical indication and spinal region. Surveys and observational studies have shown higher neuromonitoring utilization in deformity- and myelopathy-related cases, while routine single-level lumbar degenerative surgeries are frequently performed without neuromonitoring [5,6]. Systematic reviews and meta-analyses have also demonstrated that multimodal neuromonitoring techniques, including somatosensory evoked potentials, MEPs, and EMG, can improve the detection of intraoperative neurological compromise, although diagnostic accuracy varies depending on the modality and surgical context [6].

These findings indicate that, although IONM is strongly recommended for certain procedures, its use is not a universal standard of care across all spine surgeries. Accordingly, although neuromonitoring remains an important safety adjunct, clinical decision-making must also consider surgical context, anatomical factors, and the experience of the operating surgeon when unexpected circumstances arise intraoperatively.

## Case presentation

The patient was scheduled for a single-level trans-Kambin OLLIF at the L4-L5 level after failure of conservative management and concordant clinical and radiographic findings. Under standard circumstances, this procedure is performed with continuous multimodal IONM to enhance neural safety during access through Kambin’s triangle [1-6].

During anesthesia induction, administration of succinylcholine resulted in prolonged neuromuscular paralysis. Consequently, free-run EMG, triggered EMG, and MEPs were unreliable, rendering effective neuromonitoring unavailable. Cancellation of the procedure was considered; however, after a thorough multidisciplinary risk-benefit assessment, the decision was made to proceed.

Factors supporting continuation included the following: the surgery was limited to a single, uncomplicated level; preoperative imaging demonstrated favorable anatomy with a clearly defined Kambin’s triangle; and conversion to an open procedure was judged to pose greater risk, including increased blood loss and tissue disruption. The operating surgeon had more than 13 years of experience performing fluoroscopy-guided minimally invasive lumbar and trans-Kambin procedures.

In the absence of functional neuromonitoring, heightened emphasis was placed on meticulous fluoroscopic guidance and tactile feedback. Access to Kambin’s triangle was achieved using a strictly bone-referenced technique. Under continuous biplanar fluoroscopy, the probe was advanced toward the pedicle and maintained in constant contact with osseous structures, deliberately “sliding” along the pedicle and vertebral body rather than advancing freely through soft tissue. This approach minimizes the likelihood of inadvertent nerve contact by ensuring that the instrument trajectory remains constrained by bone landmarks, thereby reducing neural risk.

Care was taken to advance incrementally, confirm position frequently, and avoid aggressive manipulation. The working corridor was established only after repeated fluoroscopic confirmation of safe positioning relative to the pedicle, disc space, and exiting nerve root trajectory. Throughout the procedure, surgical progress was deliberately conservative, with continuous reassessment of anatomy and resistance, recognizing the absence of neuromonitoring as an added risk factor.

This approach reflects deliberate, experience-based surgical judgment rather than deviation from safety principles. While neuromonitoring remains an essential component of trans-Kambin OLLIF and is strongly recommended whenever feasible, this case illustrates how, under certain circumstances, safe execution may still be possible through careful patient selection, detailed anatomical knowledge, fluoroscopic precision, and surgeon experience.

Patient history and preoperative evaluation

**Figure 1:**
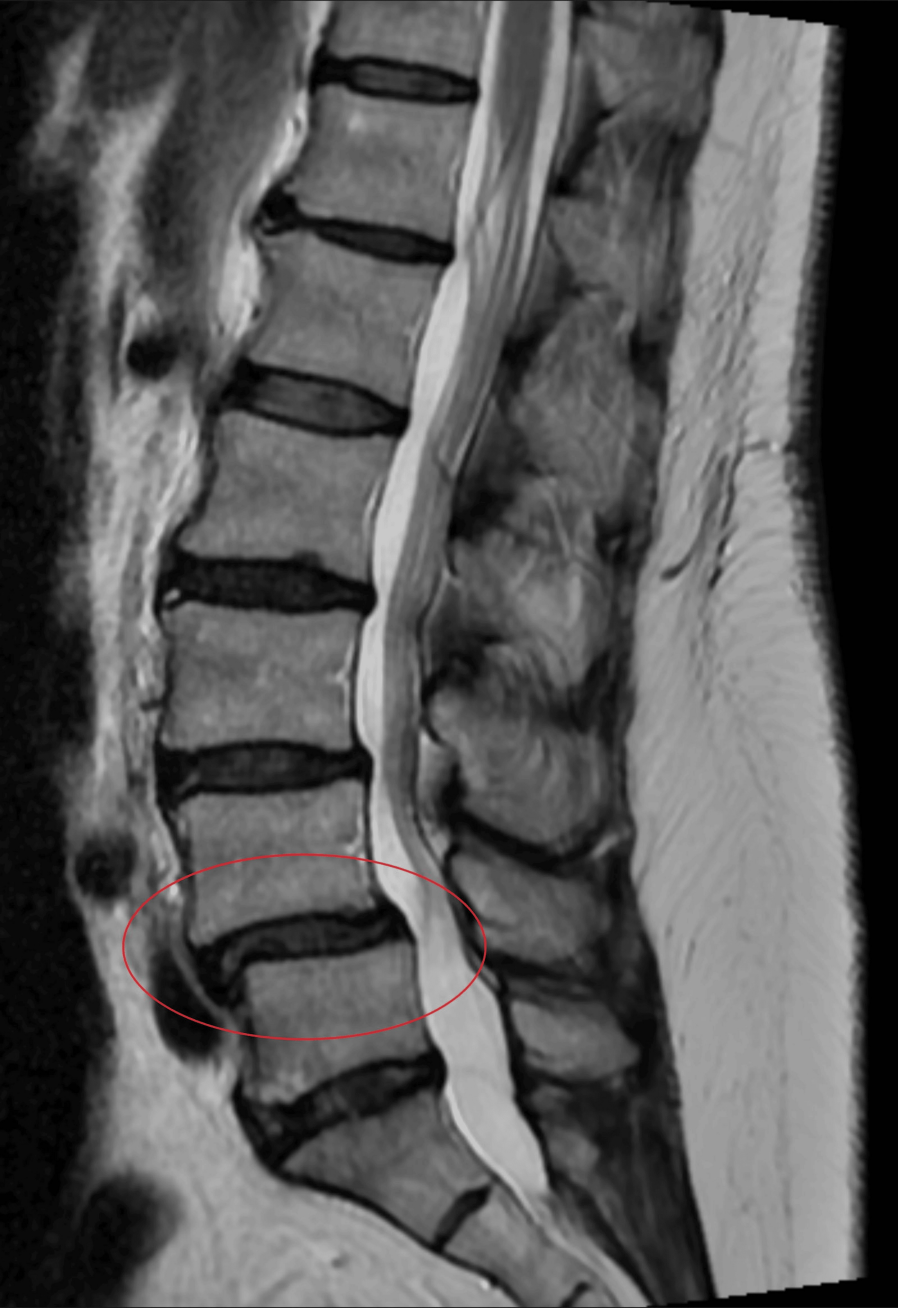
Sagittal view of lumbar MRI 10 months before surgery showing L4-L5 spondylolisthesis (circled)

**Figure 2:**
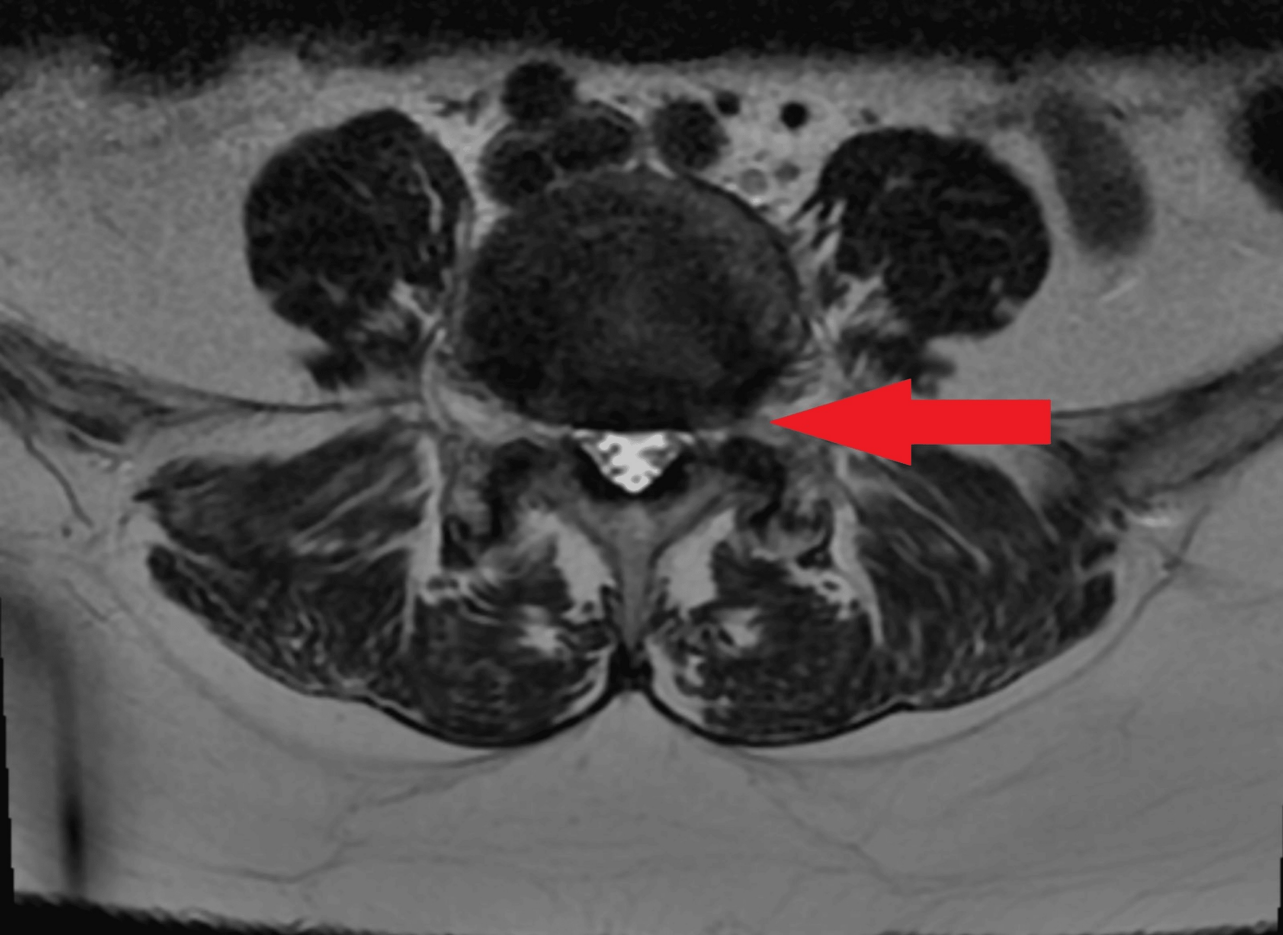
Axial view from lumbar MRI taken 10 months prior to surgery The image shows the L4-L5 disc (arrow) at the site of the spondylolisthesis.

A 57-year-old female presented with a more than 20-year history of chronic axial low back pain and bilateral lower-extremity radiculopathy. Imaging via lumbar MRI (Figure 1, Figure 2) demonstrated grade I L4-L5 spondylolisthesis with degenerative disc disease and foraminal stenosis. The patient had failed multiple forms of conservative management, including pharmacological therapy, physical therapy, injections, and activity restriction.

Relevant comorbidities included obesity (BMI 36.9) and chronic pain syndrome. There was no personal or family history of anesthesia-related complications or neuromuscular disorders, and there was no prior exposure to general anesthesia. Routine preoperative laboratory testing was unremarkable.

Anesthetic course

General anesthesia was induced using standard agents, and intubation was achieved on the first attempt with a Miller blade. Succinylcholine, a depolarizing neuromuscular blocking agent, was administered at 160 mg (20 mg/mL). Shortly after induction, train-of-four stimulation revealed no peripheral twitch response, and free-run and triggered EMG signals were unreliable. Despite adequate time for expected drug metabolism, profound neuromuscular paralysis persisted.

Given the unexpected response, the multidisciplinary team, including anesthesia, neuromonitoring, and surgery, considered aborting the procedure. However, the decision was made to proceed due to the limited single-level surgery, favorable anatomy, and anticipated higher risk with conversion to open surgery.

To our knowledge, this represents the only reported trans-Kambin OLLIF performed by the surgeon without effective neuromonitoring, highlighting the rarity and clinical significance of this case.

Surgical course

The procedure was completed in 31 minutes without intraoperative complications. Due to the absence of reliable neuromonitoring, heightened emphasis was placed on meticulous fluoroscopic guidance, strict bone-referenced technique, and continuous tactile feedback. Instrumentation and cage placement were performed incrementally under biplanar fluoroscopy, with careful attention to pedicle position, disc space trajectory, and exiting nerve root anatomy. Throughout the case, neurophysiological responses could not be elicited, even when stimulation thresholds reached 8 mA during lateral access and 35 mA at the pedicles.

Postoperative course

At the conclusion of the procedure, the patient remained profoundly paralyzed and did not regain baseline neuromuscular function. She was left intubated and sedated in the post-anesthesia care unit. After approximately two hours, weak motor responses were noted, prompting transfer to the intensive care unit for continued mechanical ventilation.

Approximately four hours after ICU transfer, the patient demonstrated intact neurological function and was able to respond appropriately to commands. She was extubated safely without complication. Given the clinical course, the anesthesiologist suspected pseudocholinesterase deficiency. Subsequent laboratory evaluation revealed a reduced pseudocholinesterase level of 2,993 U/L (reference range for females older than 42 years: 5,300-12,900 U/L).

**Figure 3:**
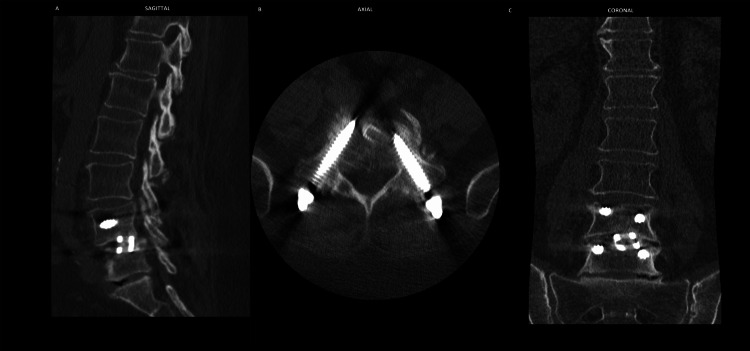
Lumbar CT five months postoperatively showing hardware condition and placement from three views (sagittal, axial, and coronal) The sagittal view (A) demonstrates placement in relation to spondylolisthesis, the axial view (B) shows alignment of the fixed pedicle screws, and the coronal view (C) displays screws and interbody in relation to each other.

The patient was discharged on postoperative day one. Imaging in the form of lumbar CT was obtained five months after the surgery to monitor her condition (Figure 3). At follow-up extending to one year and two months postoperatively, she reported complete resolution of radicular symptoms, significant improvement in axial pain, and no neurological deficits. She denied any similar anesthetic events in herself or known family members.

## Discussion

Anesthetic considerations

Prolonged neuromuscular blockade following succinylcholine administration is rare but well described [7,8]. Potential mechanisms include atypical pseudocholinesterase variants, heterozygous enzyme deficiency, or pharmacogenetic polymorphisms affecting neuromuscular junction sensitivity. In this case, laboratory findings confirmed pseudocholinesterase deficiency, which explained the prolonged paralysis and failure of neuromonitoring.

Neuromonitoring implications

IONM remains crucial for safe trans-Kambin OLLIF surgery [5,6]. This case demonstrates that neuromonitoring may become unavailable despite optimal planning and appropriate anesthetic protocols. Importantly, it does not advocate performing trans-Kambin OLLIF without neuromonitoring; rather, it provides insight into situations where deviation from standard protocols may be unavoidable.

Clinical decision-making

The decision to proceed was guided by the limited surgical scope, favorable anatomy, the surgeon’s experience, and continuous reassessment of risk. Close collaboration among the surgical, anesthesia, and neuromonitoring teams was essential to achieving a safe outcome.

## Conclusions

This case represents a rare instance of trans-Kambin OLLIF performed without effective neuromonitoring due to prolonged succinylcholine-induced neuromuscular paralysis. It is presented to highlight an exceptional circumstance and should not be interpreted as a recommendation to perform trans-Kambin OLLIF without neuromonitoring. The favorable outcome underscores the importance of understanding anesthetic variability, recognizing the limitations of neuromonitoring, and applying judicious clinical judgment in carefully selected situations. Reporting such cases contributes to a more nuanced and informed application of neuromonitoring in spine surgery while reinforcing that clinical judgment should complement, not replace, established protocols.
